# Acetanilid

**Published:** 1899-07-01

**Authors:** 


### ACETANILID.

Dr. Louis Bishop, of New York, points out that acetanilid is the basis of many of the proprietary medicines which are largely sold for the relief of pain, some of the most widely-used secret mixtures for this purpose consisting of two grains of acetanilid and half a grain each of caffein citrate and monobromate of camphor. Another useful combination, which can be prescribed in capsule, is acetanilid one grain, and quinine two grains. This is useful as an addition to other remedies in some cases of coryza. When there is suspicion of intestinal fermentation salol may be added to the acetanilid, and in certain neuroses bromide of sodium. The drug is also a useful addition to mixtures for the relief of acute indigestion attended by flatulence and distress immediately after meals. As a substitute for iodoform and other antiseptic dusting powders, acetanilid combined with boric acid is found to be most efficacious.

